# Novel mutations of the *ABCA12*, *KRT1* and *ST14* genes in three unrelated newborns showing congenital ichthyosis

**DOI:** 10.1186/s13052-022-01336-0

**Published:** 2022-08-13

**Authors:** Gregorio Serra, Luigi Memo, Paola Cavicchioli, Mario Cutrone, Mario Giuffrè, Maria Laura La Torre, Ingrid Anne Mandy Schierz, Giovanni Corsello

**Affiliations:** 1 Department of Health Promotion, Mother and Child Care, Internal Medicine and Medical Specialties “G. D’Alessandro”, University of Palermo, Palermo, Italy; 2 Clinical Genetics Outpatient Service, Serenissima Unit of Health and Social Services 3, Venice, Italy; 3 Pediatrics and Neonatal Intensive Care Unit, Angel Hospital, Mestre, Venice, Italy; 4 Pediatric Dermatology Outpatient Service, Angel Hospital, Mestre, Venice, Italy

**Keywords:** Congenital ichthyosis, Target NGS, Harlequin ichtyosis, Epidermolytic ichtyosis, Autosomal recessive ichtyosis with hypotrichosis, Case report

## Abstract

**Background:**

Congenital ichthyosis (CI) is a heterogeneous group of genetic disorders characterized by generalized dry skin, scaling and hyperkeratosis, often associated to erythroderma. They are rare diseases, with overall incidence of 6.7 in 100,000. Clinical manifestations are due to mutations in genes mostly involved in skin barrier formation. Based on clinical presentation, CI is distinguished in non-syndromic and syndromic forms. To date, mutations of more than 50 genes have been associated to different types of CI.

**Cases presentation:**

We report on three Italian unrelated newborns showing clinical signs compatible with different forms of CI of variable severity, namely Harlequin ichtyosis (HI), epidermolytic ichtyosis (EI) and autosomal recessive ichtyosis with hypotrichosis (ARIH). Target next generation sequencing (NGS) analysis identified three novel mutations of the *ABCA12*, *KRT1* and *ST14* genes, respectively associated to such congenital ichtyoses, not reported in literature. Genomic investigation allowed to provide the more appropriate management to each patient, based on an individualized approach.

**Conclusions:**

Our report highlights the wide genetic heterogeneity and phenotypic variability of CI. It expands the current knowledge on such diseases, widening their genomic database, and providing a better clinical characterization. Furthermore, it underlines the clinical relevance of NGS, which is essential to address the management of patients. Indeed, it may guide towards the most adequate approach, preventing clinical obstinacy for subjects with more severe forms and unfavorable outcomes (together with the support, in such situations, of bioethicists included within the multidisciplinary care team), as well as reassuring families in those with milder course and favorable evolution.

## Background

Congenital ichthyosis (CI) is a heterogeneous group of genetic disorders characterized by generalized dry skin, scaling and hyperkeratosis, often associated to erythroderma. They are rare diseases, with overall incidence of 6.7 in 100,000. Clinical manifestations are due to mutations in genes mostly involved in skin barrier formation. Based on clinical presentation, they are distinguished in non-syndromic and syndromic forms. The first ones are characterized by exclusive cutaneous involvement. They comprise *vulgaris* (IV, the mildest type), recessive X-linked (XRI), autosomal recessive (ARCI), and keratinopathic ichtyoses (KPI) which include epidermolytic ichtyosis (EI). ARCI encloses Harlequin ichthyosis (HI, the most severe form), lamellar ichthyosis (LI), and congenital ichthyosiform erythroderma (CIE). Conversely, in syndromic ichthyoses gene mutations may cause, besides skin alterations, abnormalities of other organs. These latter are rarer, and classified according to mode of inheritance and predominant symptoms [1–5].They include forms with prominent hair abnormalities*,* such as Netherton syndrome, and others associated with hypotrichosis, such as autosomal recessive ichtyosis with hypotrichosis (ARIH) and ichthyosis, follicular atrophoderma, hypotrichosis, and hypohydrosis (IFAH). To date, mutations of more than 50 genes have been associated to different types of CI. Such genetic heterogeneity reflects a wide phenotypic variability, ranging from severe and fatal forms to mild ones, with normal expectancy and quality of life. Target next generation sequencing (NGS) has currently significantly increased the diagnostic yield, allowing the definition of approximately 80–90% of CI cases [6, 7]. Hereby, we report on three Italian unrelated newborns showing clinical signs compatible with different types of CI of variable severity, in which NGS analysis identified the specific subtending gene defect, allowing thus the genomic diagnosis, as well as the more appropriate management based on an individualized approach.

## Cases presentation

### Patient 1

**Fig. 1:**
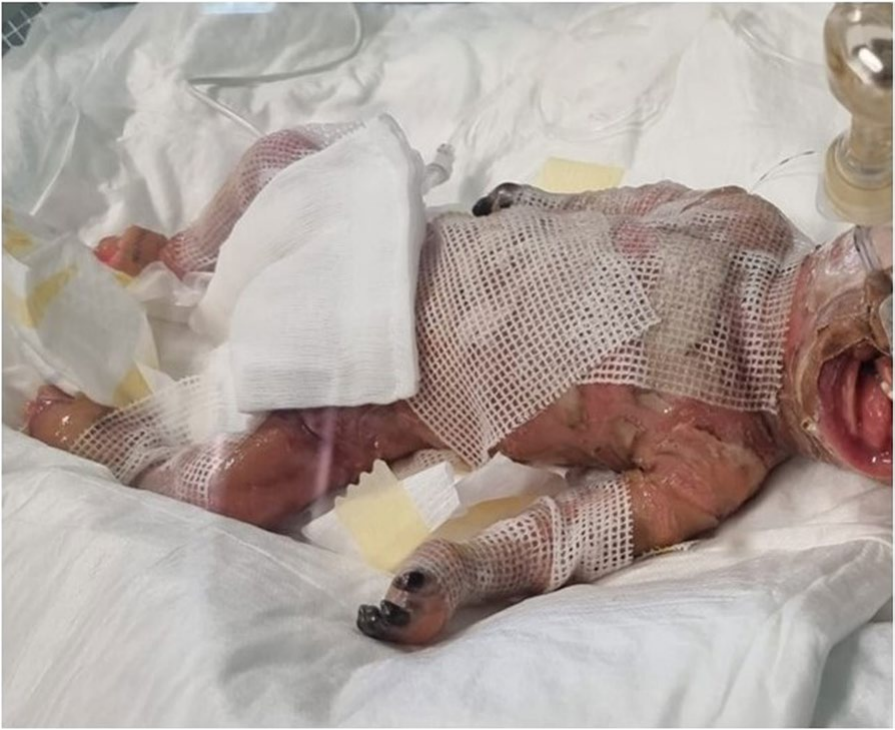
Patient 1. Hyperkeratotic plaques and diffuse hemorrhagic areas, necrosis of the fingers of both hands, generalized subcutaneous edema, pseudocontractures of the limbs (especially of fingers and toes)

A male newborn was delivered at 31^+4^ weeks of gestation (WG), due to preterm labor, by spontaneous vaginal birth. The parents were healthy and non consanguineous. Family history disclosed a brother, born prematurely at 28 WG and died for sepsis on the twelfth day of life, and a sister born prematurely as well, at 31 WG, and affected with an unspecified congenital skin disorder. Our patient had two further brothers, reported as healthy. Pregnancy was not followed either by obstetrics or other health professionals, and clinical data were thus not available. On day 2 of life he was transferred to our Department. At admission, physical examination showed large skin lesions with hyperkeratotic plaques and diffuse hemorrhagic areas, necrosis of the fingers of both hands, generalized subcutaneous edema, flattened dysplastic ears, ectropion, eclabium, macrostomia and macroglossia. Pseudocontractures of the limbs (especially of fingers and toes), resulting from cutaneous constriction, were also observed (Fig. 1). Spontaneous motility and reactivity were markedly reduced, while axial muscular tone was increased. Head and abdominal ultrasound (US) documented no abnormalities. Conversely, echocardiography showed pulmonary hypertension (pulmonic artery pressure around 35–40 mmHg), along with patent *foramen ovale*. Due to severe ectropion, ophthalmological evaluation was not carried out. Target NGS of 27 genes associated to congenital ichthyosis was performed, and identified the compound heterozygous mutations c.233_234del (p.Thr78ArgfsTer3) and c.1287 + 2_1287 + 5del of the *ABCA12* gene. Such variants were inherited from the mother and the father respectively, and them both are not reported in literature. They were then tested with Sanger sequencing, confirming the HI diagnosis (which was however available about ten days after NGS analysis was carried out), also according to the phenotype of the newborn. Postnatally, he was supported by invasive mechanical ventilation and total parenteral nutrition and later, from day 5, by minimal enteral feeding. During the first days of life blood evaluations were minimized. Laboratory tests documented hypernatremic dehydration (plasmatic sodium 153 mEq/l, chloride 122 mEq/l, and osmolarity 297 mOsm/l), which required rehydration through central venous catheter. Meanwhile, due to diffuse subcutaneous edema related to hypoproteinemia along with hypoalbuminemia, infusions with albumin, protein solution and plasma were administered. On day 6, owing to the relevant difficulties in finding venous accesses and to obtain blood samples, a femoral vein catheter was surgically positioned. In the following days, due to the worsening of hard compressive plaques on chest, forearms and legs, and based on a multidisciplinary assessment (neonatologist, pediatric surgeon, plastic surgeon, anesthesiologist and clinical geneticist), an escarotomy intervention was performed in the NICU. The following clinical course was marked by anemia, which required red blood cells transfusion, in addition to a sepsis, sustained by *Escherichia Coli* and treated with aminoglycoside antibiotic therapy. On the tenth day of life, a progressive decay of the general conditions, mainly linked to the worsening of the respiratory distress secondary to restrictive pulmonary disease, occurred. This led to lung failure, unresponsive to maximal mechanical ventilation and inotropic drugs, and finally to death.

### Patient 2

**Fig. 2:**
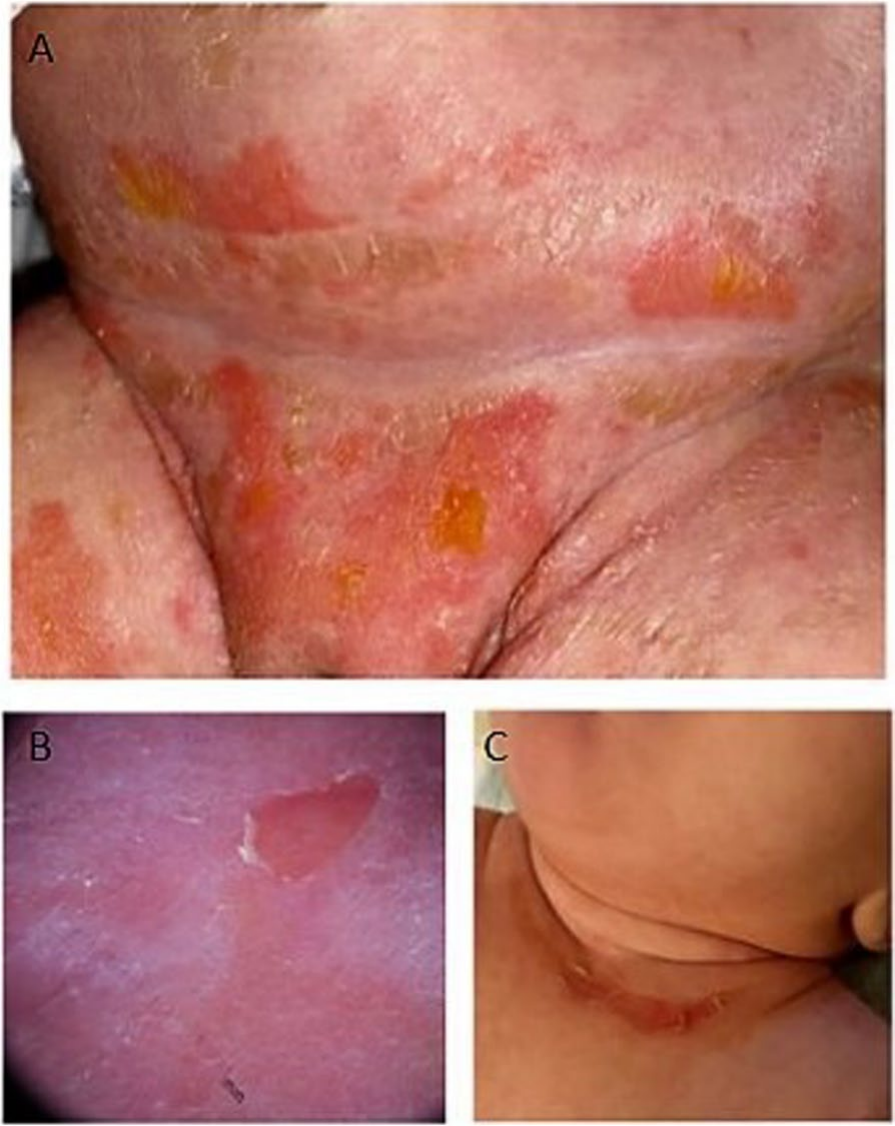
Patient 2. **A** Blisters with purulent content on lower anterior surface of the abdominal wall, pubic region, proximal segments of both thighs, and inguinal areas. **B** Small areas of erythematous skin erosions seen on dermatoscopy. **C** Mild hyperkeratosis of the anterior surface of the neck

A female newborn, delivered at 39^+1^ WG by spontaneous vaginal birth, was the second child of healthy and non consanguineous parents. Family history was negative for genetic diseases and/or genodermatoses, and also pregnancy was uneventful. On the first day of life, blisters with yellow content appeared in the lower anterior surface of the abdominal wall, pubic and perianal region, proximal segments of both thighs, and inguinal areas (Fig. 2A). Therefore, she was admitted to the Neonatology Unit. Within 48 h, the newborn developed fever, associated to generalized erythematous skin lesions with progressive bullous eruptions, in addition to further superficial blisters breaking under slight pressure. The Nikolski sign (easy separation of skin layers under application of horizontal, tangential pressure to the skin) was present. Mucosal surfaces were intact. Inspection of external genitalia showed de-epithelizing lesions in the inner and outer vulvar *labia*. Clinical and US examination of other organs and systems revealed no abnormalities. Laboratory tests showed neutrophilic leukocytosis and increased C-reactive protein, leading to the diagnostic hypothesis of a bacterial infection. In the suspicion of a staphylococcal scalded skin syndrome (SSSS), intravenous (iv) antibiotic therapy with oxacillin and gentamicin (this latter also topically applied) was empirically begun, and then changed into iv vancomycin, based on the results of skin swab culture and antibiogram (growth of an oxacillin-resistant coagulase-negative *staphylococcus hominis*). Erythroderma and the other skin lesions decreased in 10 days, and the patient was discharged on day 15, and included in a multidisciplinary (auxological and dermatological) follow-up. At age 1 month, new blisters in the gluteal region were observed, in addition to re-epithelization of the previously described lesions. At 2 months of age, after the use of protective padding, bullous lesions decreased. However, minimal desquamation, with small areas of erythematous skin erosions (seen also on dermatoscopy), were still present in the diaper region (Fig. 2B). A new skin culture, as well as blood tryptase analysis and zinc assay were performed and resulted negative, ruling out thus re-infection, mastocytosis and enteropathic acrodermatitis. Then, due to persistence of the skin lesions, it was decided to proceed in the diagnostic work-up. In the suspicion of congenital epidermolytic ichtyosis (EI) or epidermolysis bullosa (EB), target NGS analysis of the genes involved in both cutaneous diseases was performed in the patient and her parents. Genetic investigation revealed in the baby the heterozygous de novo variant c.1327A > G of the *KRT1* gene, causing the amino acid change p.Lys443Glu at the protein level, for EI diagnosis. Topical treatment with hydrating, emollient and re-lipidant creams, along with detergent oil for daily hygiene (already started after the evaluation at 1 month of age) was continued, and fusidic acid cream was added in the residual erythematous bullous lesions. The following clinical course was characterized by few minimally de-epithelizing lesions on trunk and skin folds, without other cutaneous alterations. She actually is 9 months old, and shows regular growth and development. Her general clinical conditions are good, and the skin picture is outlined by mild hyperkeratosis on the anterior surface of the neck (Fig. 2C), and on the axillary and palm-plantar regions, associated to minimal persistent bullous lesions in the diaper region. In addition, the mother reports occasional pungent odors from the hands. The previously administered topical treatment is currently continued, along with alternating use of a mild antiseptic detergent.

### Patient 3

**Fig. 3:**
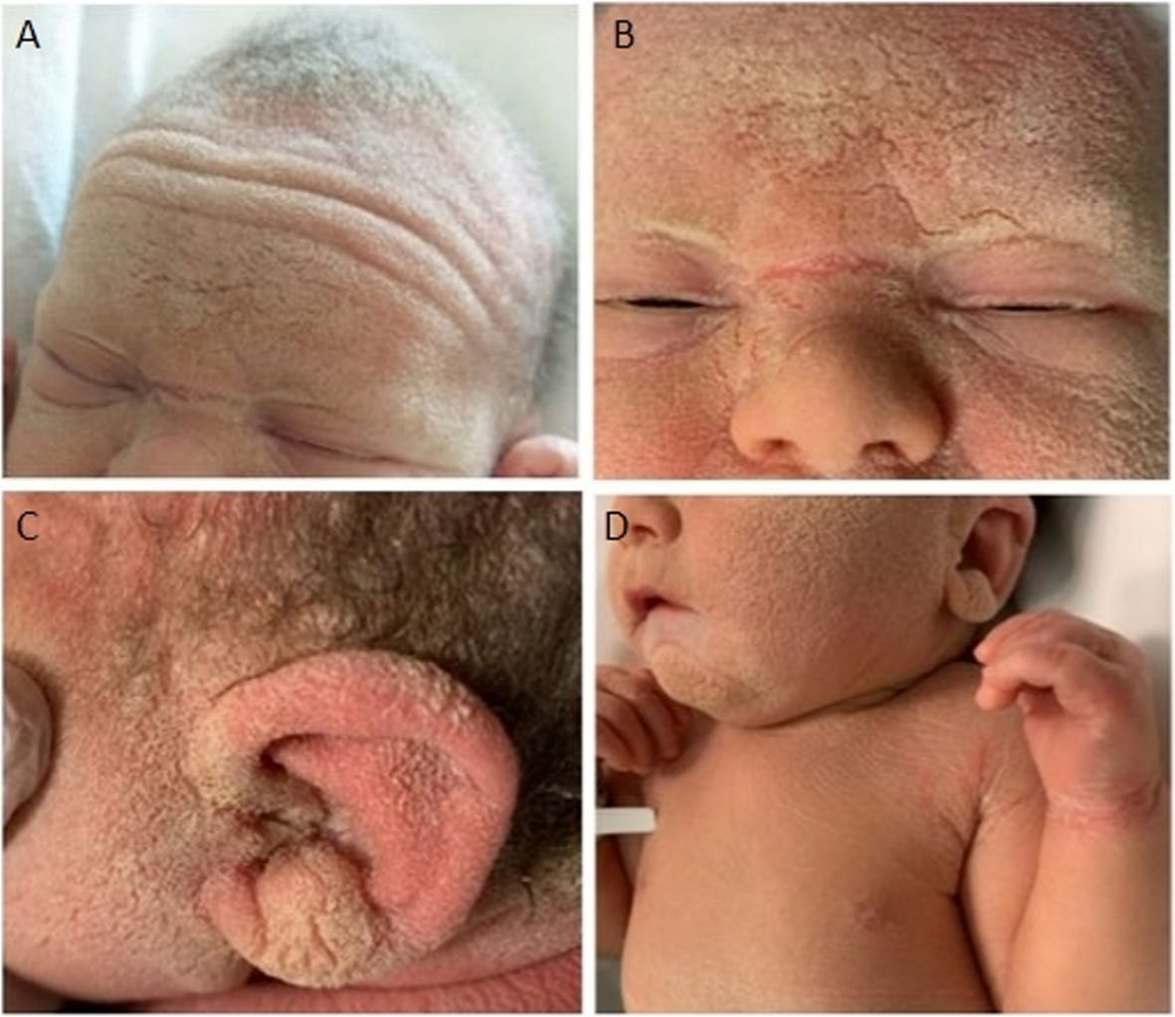
Patient 3. Diffuse thick skin, with xerosis and wide hyperkeratotic areas. **A** Sparse scalp hair (with receding front attachment) and eyebrows, cutaneous hyperlinearity of the forehead. **B** Absent eyelashes and blepharophimosis. **C** Dysplastic ear with thick helix. **D** Fissured wrist and axillary region

**Fig. 4:**
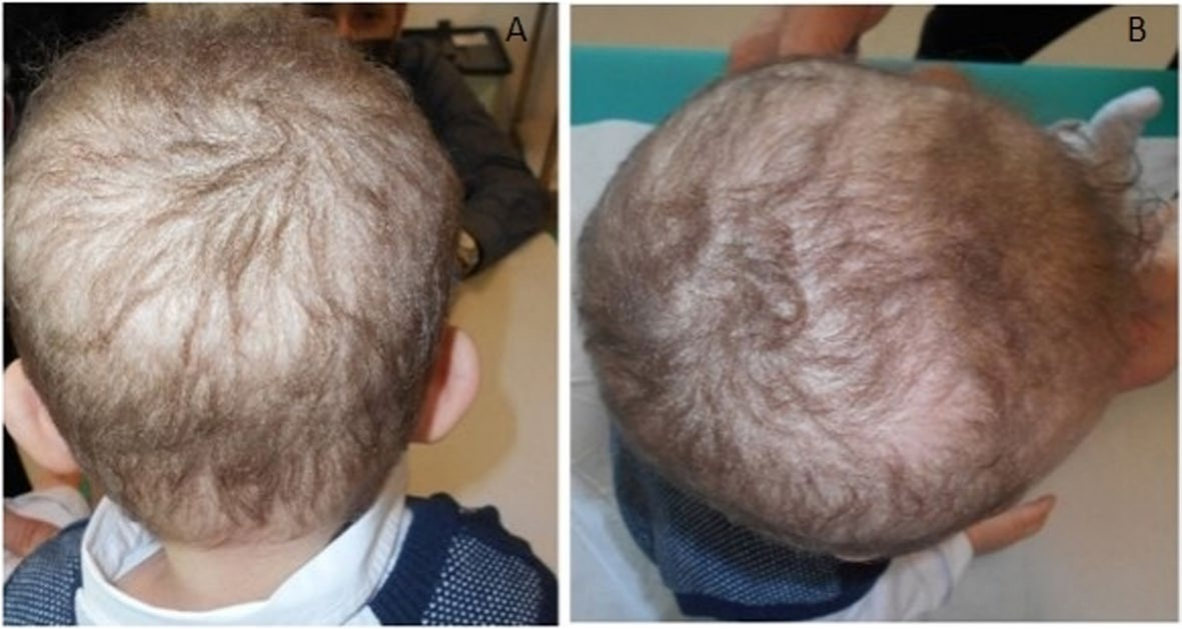
Patient 3 at age 9 months: less marked sparse hair

A male newborn was delivered at 40^+6^ WG, by cesarean section for fetal bradycardia. Parents were healthy and consanguineous (second cousins). Family history was negative for genetic diseases and/or genodermatoses, and disclosed five previous miscarriages. Current pregnancy was uneventful. At birth, physical examination showed diffuse thick skin, with xerosis and wide hyperkeratotic areas affecting scalp, face, ears, neck, chest and lower back. No collodion membrane was observed. Sparse scalp hair (with receding front attachment) and eyebrows, cutaneous hyperlinearity of the forehead, absent eyelashes and blepharophimosis, in addition to dysplastic ears with thick helices outlined the craniofacial profile. Fissured wrists, axillary regions and ankles, were also noted (Fig. 3A/B/C/D). Follicular atrophoderma, palmoplantar keratoderma, nail and mucosal changes were not observed, and sweating was normal. He was then conducted to the Neonatology Unit, to start the diagnostic work-up. Laboratory tests (including T-lymphocytes subsets and total immunoglobulin levels) and multiorgan US evaluations showed normal findings. Auditory brainstem response (ABR) documented no abnormalities, while ophthalmological assessment disclosed blepharitis. In the suspicion of congenital ichthyosis, target NGS analysis of the main associated genes found the homozygous variant c.1231G > T of *ST14*. The identification of its mutation, which leads to the introduction of the premature stop codon p.Gly411Ter, confirmed the ARIH diagnosis. Genomic analysis was then extended to parents, who were found to be heterozygous carriers of the same nonsense variant. Such mutation is not present in the database of allele frequencies (Genome Aggregation Database, gnomAD), nor it is reported in literature. The following clinical course occurred without complications. He was soon positioned into the baby incubator, with increased levels of temperature and humidity. On day 3, he was moved to the open cradle, and the clinical and laboratory monitoring for the possible appearance of fever and/or other signs of inflammation, identified no abnormalities. In the meantime, he started daily hygiene treatment with detergent oil, topical therapy with emollient/re-epithelizing creams applied on the fissured skin regions, along with gentamicin cream on those with erosion. Cleansing drops were administered in the ears, while lubricant solutions based on 0.2% sodium hyaluronate were used for the eyes. He was discharged at around 1 week in good general conditions, and included in a multidisciplinary (dermatological, auxological, ophthalmological, immunological) follow-up. At age 1 month, complete desquamation of trunk and lower region of the face were observed. Specifically, scabs with underlying erythematous skin in the frontal and periocular regions, associated to scaly plugs into the external ear canal were noted, and for which a topical treatment with 0.1% mometasone furoate cream (1 mg/g) was added. At age 4 months, the cutaneous alterations progressively improved, especially those of the face, which appeared smooth and without scales. Crusts on scalp (associated to erythematous skin compatible with seborrheic dermatitis), sacral region and lower limbs were, however, still observed. Therefore, he underwent exclusive treatment with emollient and hydrating creams, in addition to the application in the scalp of 0.1% mometasone furoate ointment. He currently is 9 months old, and shows normal growth and neuromotor development. The skin picture is outlined by remission of the previous lesions, except for minimal scaling of the scalp. Hair (Fig. 4A/B), eyelashes and eyebrows are less, but still sparse. In addition, marked photophobia, which makes him unable to keep the eyes open outdoors, followed by immediate tearing, is reported.

## Discussion and conclusions

Congenital ichthyosis refers to a large and heterogeneous group of diseases, generally monogenic, characterized by diffuse hyperkeratosis, xerosis and peeling of the skin, sometimes associated to dysmorphic features. The clinical classification includes syndromic and non-syndromic forms (Table 1). Nosology may help neonatologists in the identification of the different and extremely variable clinical phenotypes of such disorders, addressing initial diagnostic work-up and appropriate treatment. Although it is sometimes immediately possible to recognize some evident forms of ichthyosis (as in patient 1), not all clinical pictures are soon easily interpretable by the clinician. Actually, some phenotypes are not fully expressed at birth, others may show non-specific/atypical skin alterations, which may change over time (as in patient 2), or even be so mild to be initially undetected [8, 9]. Patients here reported show some features of congenital ichthyosis, which the neonatologist may find in syndromic as well non-syndromic forms. The mode of inheritance is highly important in the differential diagnosis. Among the autosomal recessive ones, a particular interest may be addressed to those with erythrodermic, scaly skin present at birth over almost the entire body surface. Within this group, HI (OMIM #242,500) is the most severe, and even lethal form. Clinical features include thick, plate-like scales, ectropion, eclabium and flattened dysplastic ears. The identification of the pathogenic mutations of *ABCA12* (OMIM #607,800) is of the utmost importance both for diagnostic confirmation of the exact genotype, and adequate prognosis evaluation and genetic counseling for family members. The *ABCA12* gene encodes a lipid transporter including ceramides, which are essential for the formation of extracellular lipid layers in the *stratum corneum* of the epidermis [10, 11]. It is the major gene involved in HI [12], and is associated to severe phenotype and high neonatal mortality rates, as occurred in our Patient 1. The variants c.233_234del (p.Thr78ArgfsTer3) and c.1287 + 2_1287 + 5del of the *ABCA12* gene, mapping on chromosome 2q35, found in our patient as compound heterozygosity, are not reported in literature. Specifically, the mutation c.233_234del is responsible for a change in the amino acid sequence of the encoded protein, starting from position 78, and causing the insertion of a premature stop codon at amino acid 81, out of a total of 2595. While, the variation c.1287 + 2_1287 + 5del is located in a canonical site of mRNA splicing. Then, in accordance with the American College of Medical Genetics (ACMG) recommendations, such mutations have been considered pathogenic [13]. Actually, compound heterozygosity (loss of function or missense) for two different alleles is associated to milder phenotype and better prognosis. This may be justified by a possible compensatory effect of different mutations affecting the two alleles (maternal and paternal) of the same gene [14]. However, in our Patient 1 compound heterozygosity led to a severe phenotype and poor prognosis, with early fatal outcome occurring in neonatal age. This may be explained by the nature of both mutations significantly affecting protein synthesis, with effects on splicing mechanisms, and relevant quantitative reduction due to very early formation of a stop codon.

**Table 1:** Clinical classification of congenital ichtyosis (modified by Oji V. et al., 2010) [4]

Non-syndromic forms	Syndromic forms
**Common ichthyoses**	**X-linked ichthyosis syndromes**
Ichthyosis vulgaris	Recessive X-linked ichthyosis
Recessive X-linked ichthyosis non-syndromic presentation	Ichthyosis follicularis-atrichia-photophobia syndrome
**Autosomal recessive congenital ichthyosis**	Conradi-Hünermann-Happle syndrome
*Major types*	**Autosomal ichthyosis syndromes (with) Prominent hair abnormalities**
Harlequin ichthyosis	Netherton syndrome
Lamellar ichthyosis	Ichthyosis hypotrichosis syndrome
Congenital ichthyosiform erythroderma	Ichthyosis-hypotrichosis-sclerosing cholangitis syndrome
*Minor variants*	Trichothiodystrophy
Self-healing collodion baby	**Prominent neurologic signs**
Acral self-healing collodion baby	Sjögren-Larsson syndrome
Bathing suit ichthyosis	Refsum syndrome
**Keratinopathic ichthyosis**	Mental retardation-enteropathy-deafness-neuropathy-ichthyosis-keratodermia syndrome
*Major types*	**Severe evolution**
Epidermolytic ichthyosis	Gaucher syndrome type 2
Superficial epidermolytic ichthyosis	Multiple sulfatase deficiency
*Minor variants*	Cerebral dysgenesis-neuropathy-ichthyosis-palmoplantar keratoderma syndrome
Annular epidermolytic ichthyosis	Arthrogryposis-renal dysfunction-cholestasis syndrome
Ichthyosis Curth-Macklin	**Other associated signs**
Autosomal recessive epidermolytic ichthyosis	Keratitis-ichthyosis-deafness syndrome
Epidermolytic nevi	Neutral lipid storage disease with ichthyosis
**Other forms**	Ichthyosis prematurity syndrome
Loricrin keratoderma	
Erythrokeratodermia variabilis	
Peeling skin disease	
Congenital reticular ichthyosiform erythroderma	
Keratosis linearis-ichthyosis congenita-keratoderma	

Keratinopathic ichtyosis (KPI) is a group of non-syndromic CI, and represents a family of superficial keratin keratodermas including epidermolytic ichtyosis (EI, OMIM #607,602), superficial epidermolytic ichthyosis (SEI) and other forms. Among them, EI is the most frequent type. It is caused by heterozygous mutations of the keratin genes *KRT1* (OMIM #139,350) or *KRT10* (OMIM #148,080), and shows autosomal dominant transmission, although approximately 50% of cases occur de novo (as in our Patient 2) [15]. Mutations in such genes, encoding for epidermal suprabasal keratinocytes (keratin 1 or 10), cause epithelial barrier disruption with high risk of infection and electrolyte imbalances. At birth, patients usually have diffuse non-ichthyosiform erythroderma with multiple erosions, which may be associated to blisters, and scaly and exfoliated skin. In our Patient 2, it was necessary to administer specific antibiotic therapy for the onset of a cutaneous infection, documented by skin swab positivity for oxacillin-resistant staphylococcus. Erythroderma and blisters decrease over time, and hyperkeratosis, sometimes involving scalp (with alopecia) and neck, occurs in the first few months of life (as also observed in our Patient 2). After the neonatal stage, blisters and intermittent skin infections can appear, and be responsible for disfigurement, pungent odors (present also in our case) and consequent possible severe psychological and social discomfort [16, 17]. Treatment is symptomatic, and aimed at preventing or decreasing the risk of dehydration, electrolyte imbalances and skin superinfections and/or sepsis. It includes topical emollients, protective padding, and keratolytic agents. Differential diagnosis includes skin disorders (EB, ichthyosis *bullosa* of Siemens, ichthyosis *hystrix*, and peeling skin syndrome), genetic diseases causing neonatal blistering (i.e., *incontinentia pigmenti*, and Ankyloblepharon–Ectodermal defects–Clef lip/palate syndrome) [18], and other conditions acquired early in life (as SSSS, bullous *impetigo*, Stevens–Johnson syndrome, and toxic epidermal necrolysis). In our Patient 2, target NGS revealed a novel variant of the *KRT1* gene, mapping on chromosome 12q13.13, not reported in literature.

Autosomal recessive ichthyosis with hypotrichosis (ARIH) syndrome (OMIM #602,400) is characterized by vernix-like scaling skin at birth, with the succession of generalized white, fine scales with scalp involvement and sparing of flexural surfaces, along with diffuse, non-scarring hypotrichosis. The scalp hair is fair, sparse and curly. Eye involvement includes photophobia and/or blepharitis. *ST14* gene mutations are associated to ARIH, and were found in Patient 3. Such gene encodes a type II transmembrane serine protease of the S1 trypsin-like protein family [19], known as matriptase or ST14, and involved in the degradation of the cellular matrix [20]. It is expressed in various tissues, including thymic epithelium and stroma, and plays a crucial role in the normal development of epidermis (*stratum corneum* and hair follicles) and immune system. ARIH and IFAH have been observed in families with high degree of inbreeding (of Arab, Turkish, Romanian, Israelian, Bedouin and Pakistani origin) [21]. To date, there is no clear genotype–phenotype correlation between the different *ST14* mutations and the severity of the clinical picture. In our Patient 3, a novel mutation of the *ST14* gene (OMIM #606,797), mapping on chromosome 11q24.3, was found.

Target NGS has been successfully applied to rare diseases with genetic heterogeneity, overlapping phenotypes and with causal genes involved in common pathogenic pathways [22]. Patients with congenital ichthyosis often remain without a clear genotypic diagnosis [23]. We found three novel mutations of the *ABCA12*, *KRT1* and *ST14* genes associated to different forms of CI, namely Harlequin ichtyosis, epidermolytic ichtyosis and autosomal recessive ichtyosis with hypotrichosis, respectively, showing a wide spectrum of clinical manifestations with variable severity and evolution.

Our report highlights the relevant genetic heterogeneity and phenotypic variability of CI. It underlines how for neonatologists and pediatricians may be crucial to achieve the molecular characterization of CI, similarly to other congenital genetic diseases, to make a correct and more precise prognosis evaluation, and to allow proper management based on an individualized approach, as well as adequate genetic counseling [9, 24, 25]. This latter, which includes discussion of potential risks of transmission to offspring and reproductive options, should be offered, if possible before pregnancy, to young adults who are affected or at risk. Once a pathogenic variant has been identified in an affected (autosomal dominant diseases) and/or carrier (autosomal recessive forms) family member, prenatal testing (through chorionic villus sampling and/or amniocentesis), for a pregnancy at increased risk (e.g. an index case among relatives), and preimplantation genetic diagnosis are possible. Our study expands the current knowledge on CI, widening the genomic database and providing a better characterization of these conditions. Target NGS may aid clinicians during the diagnostic process (e.g., avoiding invasive methods like skin biopsy), and also later adapting the clinical management to the specific case [26–29]. Indeed, it may address towards the most appropriate approach, preventing clinical obstinacy in conditions burdened by serious complications or at high risk of death (as in our Patient 1). However, in such situations the support of bioethicists within the multidisciplinary care team may help in determining the adequate level of aggressive medical interventions. In addition, genomic sequencing may conversely reassure families in cases with good clinical evolution, also due to adequate control or limitation of the adverse outcomes (even anticipating them, as in Patients 2 and 3) of the disease with appropriate therapies.

## Data Availability

The datasets used and analyzed during the current study are available from the corresponding author on reasonable request.

## Abbreviations

ABR: Auditory brainstem response
ACMG: American College of Medical Genetics
ARCI: Autosomal recessive congenital ichthyosis
ARIH: Autosomal recessive ichthyosis with hypotrichosis
CI: Congenital ichthyosis
CIE: Congenital ichthyosiform erythroderma
EB: Epidermolysis bullosa
EI: Epidermolytic ichthyosis
gnomAD: Genome Aggregation Database
HI: Harlequin ichthyosis
IFAH: Ichthyosis, follicular atrophoderma, hypotrichosis and hypohydrosis
IV: Ichthyosis vulgaris
iv: Intravenous
KPI: Keratinopathic ichtyoses
LI: Lamellar ichthyosis
NGS: Next generation sequencing
NICU: Neonatal intensive care unit
OFC: Occipitofrontal circumference
SD: Standard deviation
SEI: Superficial epidermolytic ichthyosis
SSSS: Staphylococcal scalded skin syndrome
US: Ultrasound
WG: Weeks of gestation

## References

[1] Israeli S, Goldberg I, Fuchs-Telem D, Bergman R, Indelman M, Bitterman-Deutsch O, Harel A, Mashiach Y, Sarig O, Sprecher E (2013). Non-syndromic autosomal recessive congenital ichthyosis in the Israeli population. Clin Exp Dermatol.

[2] Takeichi T, Akiyama M (2016). Inherited ichthyosis: Non-syndromic forms. J Dermatol.

[3] Yoneda K (2016). Inherited ichthyosis: Syndromic forms. J Dermatol.

[4] Oji V, Tadini G, Akiyama M, Blanchet Bardon C, Bodemer C, Bourrat E, Coudiere P, DiGiovanna JJ, Elias P, Fischer J, Fleckman P, Gina M, Harper J, Hashimoto T, Hausser I, Hennies HC, Hohl D, Hovnanian A, Ishida-Yamamoto A, Jacyk WK, Leachman S, Leigh I, Mazereeuw-Hautier J, Milstone L, Morice-Picard F, Paller AS, Richard G, Schmuth M, Shimizu H, Sprecher E, Van Steensel M, Taïeb A, Toro JR, Vabres P, Vahlquist A, Williams M, Traupe H (2010). Revised nomenclature and classification of inherited ichthyoses: results of the First Ichthyosis Consensus Conference in Sorèze 2009. J Am Acad Dermatol.

[5] Vahlquist A, Fischer J, Törmä H (2018). Inherited Nonsyndromic Ichthyoses: An Update on Pathophysiology, Diagnosis and Treatment. Am J Clin Dermatol.

[6] Mazereeuw-Hautier J, Vahlquist A, Traupe H, Bygum A, Amaro C, Aldwin M, Audouze A, Bodemer C, Bourrat E, Diociaiuti A, Dolenc-Voljc M, Dreyfus I, El Hachem M, Fischer J, Gånemo A, Gouveia C, Gruber R, Hadj-Rabia S, Hohl D, Jonca N, Ezzedine K, Maier D, Malhotra R, Rodriguez M, Ott H, Paige DG, Pietrzak A, Poot F, Schmuth M, Sitek JC, Steijlen P, Wehr G, Moreen M, O'Toole EA, Oji V, Hernandez-Martin A (2019). Management of congenital ichthyoses: European guidelines of care, part one. Br J Dermatol.

[7] Fischer J, Bourrat E (2020). Genetics of Inherited Ichthyoses and Related Diseases. Acta Derm Venereol.

[8] Dyer JA, Spraker M, Williams M (2013). Care of the newborn with ichthyosis. Dermatol Ther.

[9] Vahlquist A, Törmä H (2020). Ichthyosis: A Road Model for Skin Research. Acta Derm Venereol..

[10] Ahmed H, O'Toole EA (2014). Recent advances in the genetics and management of harlequin ichthyosis. Pediatr Dermatol..

[11] Akiyama M (2011). The roles of ABCA12 in keratinocyte differentiation and lipid barrier formation in the epidermis. Dermatoendocrinol.

[12] Thomas AC, Cullup T, Norgett EE, Hill T, Barton S, Dale BA, Sprecher E, Sheridan E, Taylor AE, Wilroy RS, DeLozier C, Burrows N, Goodyear H, Fleckman P, Stephens KG, Mehta L, Watson RM, Graham R, Wolf R, Slavotinek A, Martin M, Bourn D, Mein CA, O'Toole EA, Kelsell DP (2006). ABCA12 is the major harlequin ichthyosis gene. J Invest Dermatol.

[13] Richards S, Aziz N, Bale S, Bick D, Das S, Gastier-Foster J, Grody WW, Hegde M, Lyon E, Spector E, Voelkerding K, Rehm HL, ACMG Laboratory Quality Assurance Committee (2015). Standards and guidelines for the interpretation of sequence variants: a joint consensus recommendation of the American College of Medical Genetics and Genomics and the Association for Molecular Pathology. Genet Med.

[14] Akiyama M (2010). ABCA12 mutations and autosomal recessive congenital ichthyosis: a review of genotype/phenotype correlations and of pathogenetic concepts. Hum Mutat.

[15] Hotz A, Oji V, Bourrat E, Jonca N, Mazereeuw-Hautier J, Betz RC, Blume-Peytavi U, Stieler K, Morice-Picard F, Schönbuchner I, Markus S, Schlipf N, Fischer J (2016). Expanding the Clinical and Genetic Spectrum of KRT1, KRT2 and KRT10 Mutations in Keratinopathic Ichthyosis. Acta Derm Venereol.

[16] Chamcheu JC, Siddiqui IA, Syed DN, Adhami VM, Liovic M, Mukhtar H (2011). Keratin gene mutations in disorders of human skin and its appendages. Arch Biochem Biophys.

[17] Rimoin L, Graham JM (2012). Blistering skin disorders in the neonate. Clin Pediatr (Phila).

[18] Serra G, Antona V, Giuffré M, Li Pomi F, Lo Scalzo L, Piro E, Schierz IAM, Corsello G (2021). Novel missense mutation of the TP63 gene in a newborn with Hay-Wells/Ankyloblepharon-Ectodermal defects-Cleft lip/palate (AEC) syndrome: clinical report and follow-up. Ital J Pediatr.

[19] Hooper JD, Clements JA, Quigley JP, Antalis TM (2001). Type II transmembrane serine proteases. Insights into an emerging class of cell surface proteolytic enzymes. J Biol Chem.

[20] Lin CY, Anders J, Johnson M, Sang QA, Dickson RB (1999). Molecular cloning of cDNA for matriptase, a matrix-degrading serine protease with trypsin-like activity. J Biol Chem.

[21] Ahmad F, Ahmed I, Nasir A, Umair M, Shahzad S, Muhammad D, Santos-Cortez RLP, Leal SM, Ahmad W (2018). A disease-causing novel missense mutation in the ST14 gene underlies autosomal recessive ichthyosis with hypotrichosis syndrome in a consanguineous family. Eur J Dermatol.

[22] Nardello R, Plicato G, Mangano GD, Gennaro E, Mangano S, Brighina F, Raieli V, Fontana A (2020). Two distinct phenotypes, hemiplegic migraine and episodic Ataxia type 2, caused by a novel common CACNA1A variant. BMC Neurol.

[23] Liu Z, Zhu L, Roberts R, Tong W (2019). Toward Clinical Implementation of Next-Generation Sequencing-Based Genetic Testing in Rare Diseases: Where Are We?. Trends Genet.

[24] Fioretti T, Auricchio L, Piccirillo A, Vitiello G, Ambrosio A, Cattaneo F, Ammendola R, Esposito G (2020). Multi-Gene Next-Generation Sequencing for Molecular Diagnosis of Autosomal Recessive Congenital Ichthyosis: A Genotype-Phenotype Study of Four Italian Patients. Diagnostics (Basel).

[25] Serra G, Antona V, Giuffrè M, Piro E, Salerno S, Schierz IAM, Corsello G (2022). Interstitial deletions of chromosome 1p: novel 1p31.3p22.2 microdeletion in a newborn with craniosynostosis, coloboma and cleft palate, and review of the genomic and phenotypic profiles. Ital J Pediatr..

[26] Piro E, Schierz IAM, Antona V, Pappalardo MP, Giuffrè M, Serra G, Corsello G (2020). Neonatal hyperinsulinemic hypoglycemia: case report of kabuki syndrome due to a novel KMT2D splicing-site mutation. Ital J Pediatr.

[27] Serra G, Corsello G, Antona V, D'Alessandro MM, Cassata N, Cimador M, Giuffrè M, Schierz IAM, Piro E (2020). Autosomal recessive polycystic kidney disease: case report of a newborn with rare PKHD1 mutation, rapid renal enlargement and early fatal outcome. Ital J Pediatr.

[28] Piro E, Nardello R, Gennaro E, Fontana A, Taglialatela M, Mangano GD, Corsello G, Mangano S (2019). A novel mutation in *KCNQ3*-related benign familial neonatal epilepsy: electroclinical features and neurodevelopmental outcome. Epileptic Disord.

[29] Serra G, Memo L, Coscia A, Giuffré M, Iuculano A, Lanna M, Valentini D, Contardi A, Filippeschi S, Frusca T, Mosca F, Ramenghi LA, Romano C, Scopinaro A, Villani A, Zampino G, Corsello G, their respective Scientific Societies and Parents’ Associations (2021). Recommendations for neonatologists and pediatricians working in first level birthing centers on the first communication of genetic disease and malformation syndrome diagnosis: consensus issued by 6 Italian scientific societies and 4 parents' associations. Ital J Pediatr.

